# Painless vertebral artery dissection mimicking benign paroxysmal positional vertigo: a case report

**DOI:** 10.1097/MS9.0000000000005379

**Published:** 2026-07-28

**Authors:** Sobin Pant, Dibij Adhikari, Sachet Subedi, Raju Pandit, Jayaram Lamichhane, Bikram Prasad Gajurel

**Affiliations:** a Department of Neurology, Maharajgunj Medical Campus, Institute of Medicine, Tribhuvan University, Kathmandu, Nepal; b Department of Neurology, Tribhuvan University Teaching Hospital, Kathmandu, Nepal

**Keywords:** BPPV, neurology, posterior circulation stroke, vertebral artery dissection

## Abstract

Acute positional vertigo is a common neurological presentation, which is often assumed to be due to benign peripheral vestibular causes. This paper presents the case of a woman in her late 30s who presented with brief, recurrent, position-triggered vertigo. She had no headache, neck pain, trauma history, or vascular risk factors. The clinical history closely resembled benign paroxysmal positional vertigo. However, neurological examination revealed subtle central signs: internuclear ophthalmoplegia and cerebellar ataxia. Brain imaging demonstrated infarction in the left cerebellar hemisphere. Brainstem imaging showed no corresponding abnormalities. Vascular imaging revealed extracranial left vertebral artery dissection. The patient condition was managed conservatively with antiplatelet therapy and a statin. Her symptoms resolved spontaneously, with no recurrence of vertigo on follow-up. This case illustrates that vertebral artery dissection may present without pain and highlights the importance of careful neurological examination in patients with acute vertigo.

## Introduction

Dizziness and vertigo account for approximately 3–4% of all emergency department (ED) presentations, representing a common but diagnostically challenging complaint for frontline clinicians[1]. While most cases arise from benign peripheral vestibular disorders, a small but clinically important minority arise from posterior circulation stroke (PCS)[2]. The diagnostic challenge is greatest when the history suggests benign paroxysmal positional vertigo (BPPV) – brief, position-triggered vertigo – while subtle central signs indicate a more serious etiology[3].

Central cerebellar lesions may produce paroxysmal position-dependent vertigo, a presentation sometimes termed central paroxysmal positional vertigo, and may closely mimic peripheral vestibulopathy ^[^4–6^]^ In young adults, cervical artery dissection is the leading vascular cause of PCS, with vertebral artery dissection (VAD) representing a key etiology of cerebellar and brainstem infarction[7]. Classically, vertebral artery dissection is associated with head or neck pain and delayed ischemic deficits. However, nearly one in four patients may present without craniocervical pain, which can contribute to delayed or missed diagnoses[8].

HIGHLIGHTS

- Painless vertebral artery dissection may present as isolated positional vertigo.
- Central causes of vertigo can closely mimic benign paroxysmal positional vertigo.
- Subtle ocular motor findings are key to identifying vascular vertigo.
- Posterior circulation stroke may be present despite a normal brainstem MRI.
- Careful neurological examination is essential in young patients with acute vertigo.

## Case presentation

A previously healthy female in her late 30s presented to the neurology department with a 1-week history of repetitive episodes of sudden-onset vertigo. The patient reported that the symptoms were precipitated by head movement and lasted only a few seconds before resolving. These episodes were not associated with nausea, vomiting, tinnitus, or hearing loss.

There was no history of diplopia, dysarthria, dysphagia, or other focal neurological deficits. The patient denied any history of recent trauma, neck manipulation, or cervical pain. She had no traditional cerebrovascular risk factors, such as smoking, hyperlipidemia, diabetes mellitus, hypertension, coronary artery disease, or atrial fibrillation.

Upon examination, vital signs were within normal limits, and higher mental functions were intact. Examination of the cranial nerves revealed a specific deficit: difficulty in left eye adduction with dissociated nystagmus of the abducting right eye, consistent with left internuclear ophthalmoplegia. There was no spontaneous or gaze-evoked nystagmus. Sensory and motor examinations were unremarkable. However, cerebellar testing revealed mild impairments in tandem walking and a finger-to-nose test on the left side. She had a mildly wide-based gait with postural stability. In view of central ocular motor signs and cerebellar signs, positional maneuvers (e.g., Dix-Hallpike) were deferred to avoid potential neck manipulation in the setting of a suspected central vascular event. There were no features of autonomic dysfunction.

**Figure 1.**
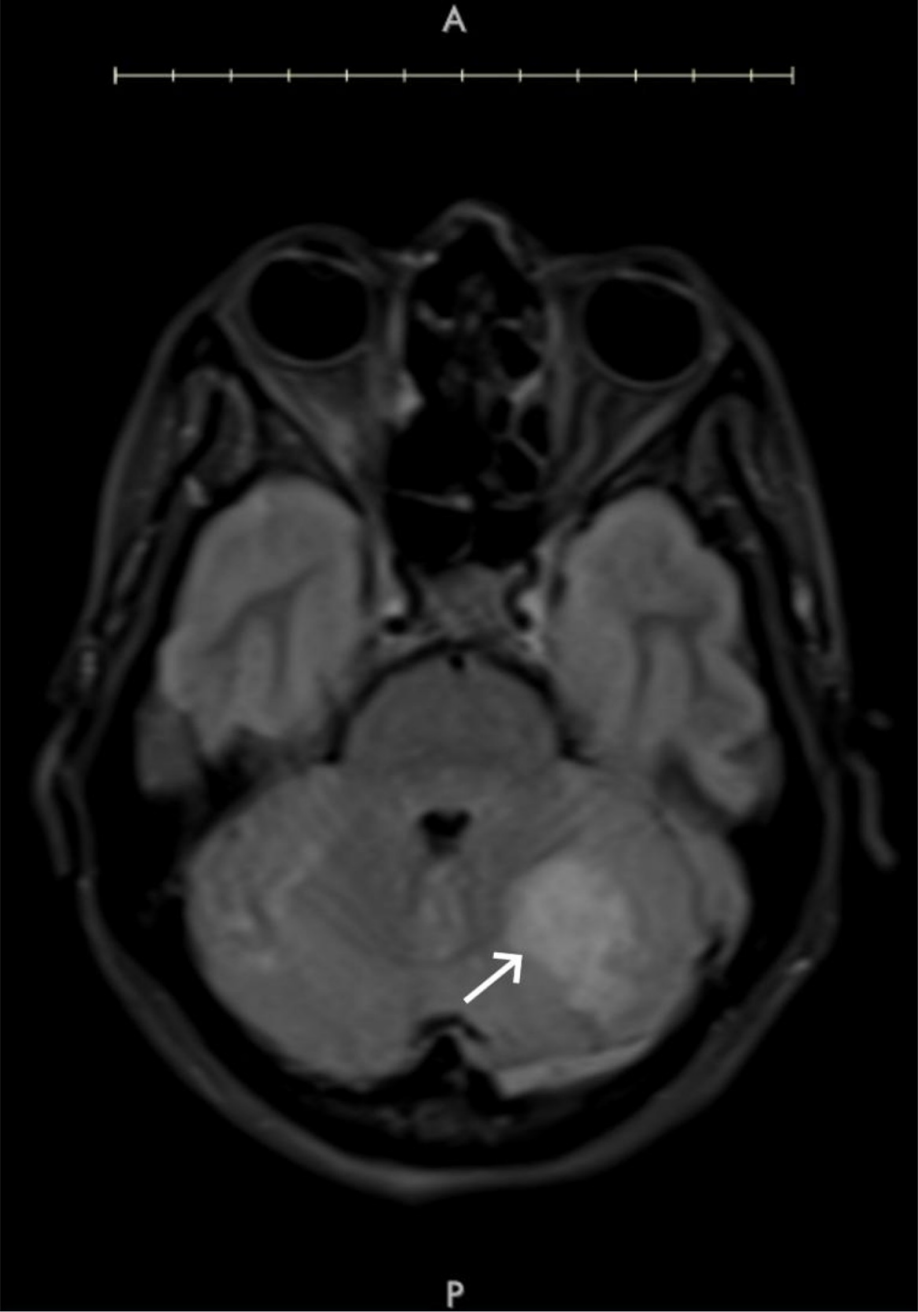
Axial fluid-attenuated inversion recovery magnetic resonance image of the brain demonstrates a hyperintense lesion in the left cerebellar hemisphere (arrow), consistent with a subacute cerebellar infarction.

Brain Magnetic Resonance Imaging revealed a subacute infarct involving the left cerebellar hemisphere. On fluid-attenuated inversion recovery (FLAIR) sequences, the lesion appeared hyperintense, corresponding to an area of restricted diffusion on diffusion-weighted imaging (Fig. 1). Notably, despite the clear clinical findings of internuclear ophthalmoplegia, no ischemic signal changes were observed in the brainstem (pons or midbrain).

A workup for stroke in the young was initiated. To rule out a hypercoagulable state, antinuclear antibody and antiphospholipid antibody tests were conducted; both yielded negative results. Routine biochemical and hematological profiles were within normal limits. Cardiac evaluation, including echocardiography and Holter monitoring, revealed no abnormalities.

**Figure 2.**
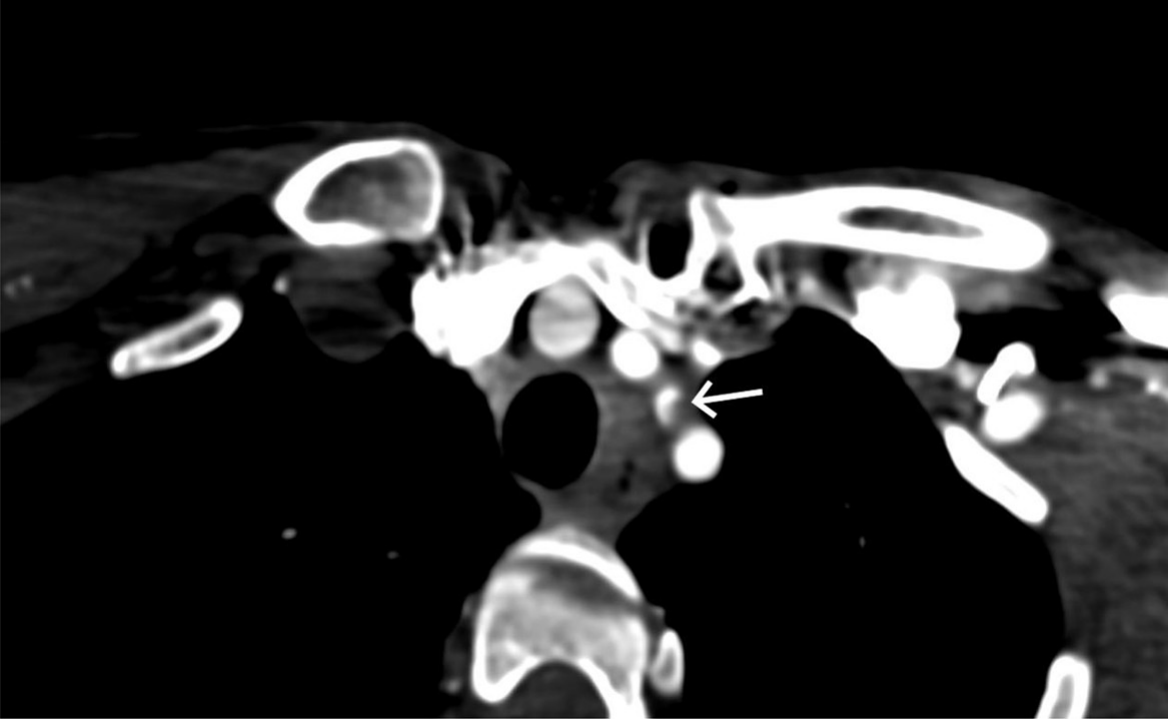
Axial computed tomography angiography image of the neck, demonstrating a partially occlusive intraluminal filling defect within the V1 segment of the left vertebral artery (arrow), consistent with vertebral artery dissection.

**Figure 3.**
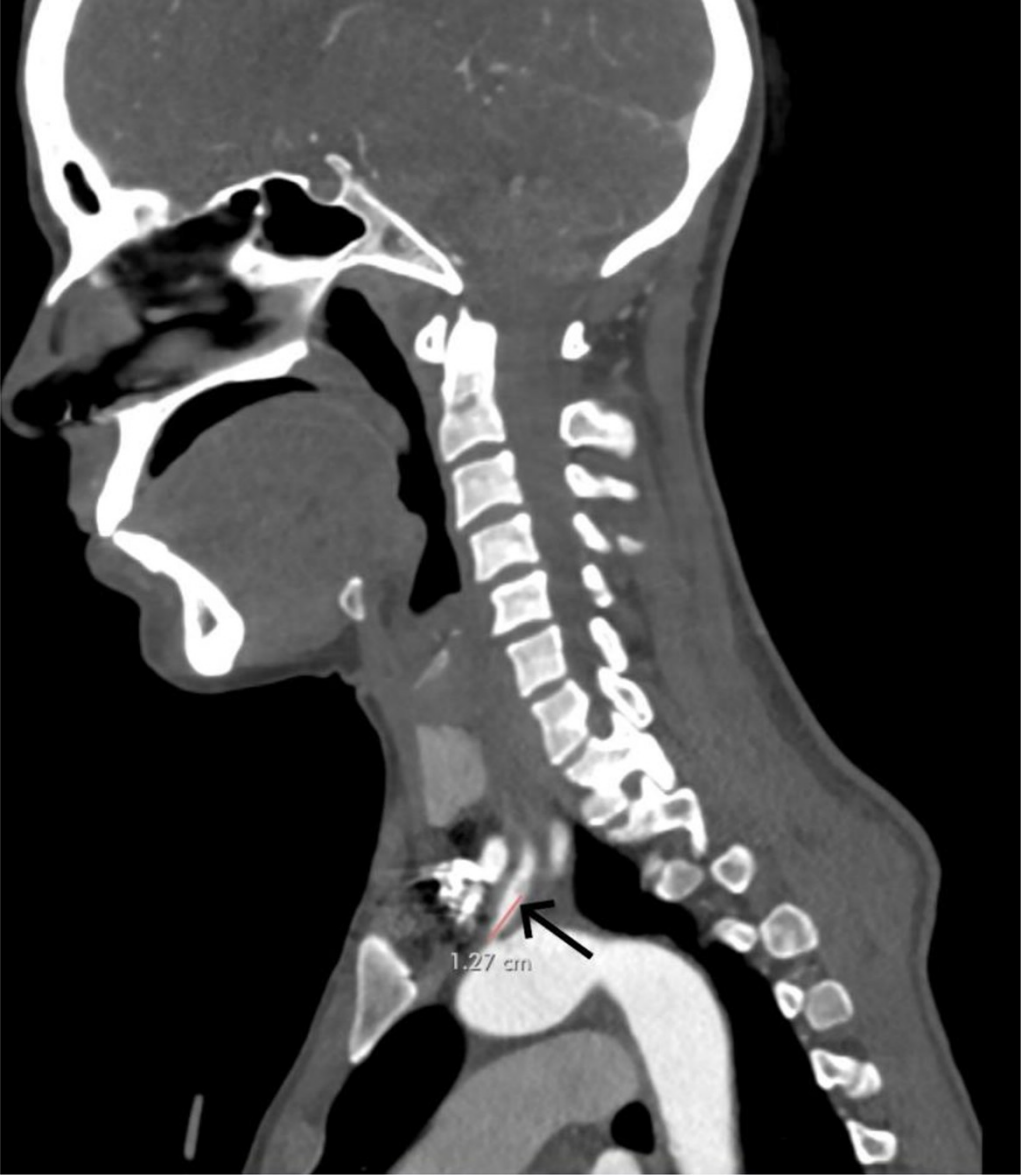
Sagittal computed tomography angiography image of the neck demonstrating luminal irregularity of the extracranial left vertebral artery with a linear intimal flap (arrow) approximately 1.27 cm from its origin, consistent with vertebral artery dissection.

Computed Tomography Angiography of the neck vessels was performed to investigate the vascular etiology. This revealed a small dissection flap with an eccentric, partially occlusive mural thrombus in the V1 segment of the left vertebral artery, resulting in approximately 30% luminal narrowing (Figs 2 and 3). The remaining vertebral and basilar artery segments were normal. The patient was medically managed with a single antiplatelet agent (aspirin 100 mg daily) and high-intensity statin therapy (atorvastatin 40 mg daily), along with supportive care.

The patient showed significant clinical improvement during hospitalization. Her symptoms resolved completely, and she was discharged after 11 days with no residual neurological deficits. At the 4-week follow-up, the patient remained asymptomatic with no recurrence of vertigo. Follow-up was arranged every three months, with repeat computed tomography angiography planned at 6 months to assess vessel healing, recanalization, or potential pseudoaneurysm formation.

## Discussion

This case illustrates how vertebral artery dissection may initially mimic benign paroxysmal positional vertigo based on clinical history. However, the findings of central neurological signs, including internuclear ophthalmoplegia and cerebellar ataxia, supported a central cause. Central paroxysmal positional vertigo is a well-documented pitfall, with emergency department misdiagnosis rates as high as 50%[4].

Central Paroxysmal Positional Vertigo has been associated with lesions involving the nodulus and uvula of the inferior cerebellar vermis^[^4,5^]^. These structures are supplied by the medial branch of the posterior inferior cerebellar artery (PICA)^[^5,6^]^. In our patient, a left posterior inferior cerebellar artery-territory infarct was demonstrated on MRI and may explain the cerebellar signs, including limb ataxia. Although the precise mechanism cannot be established definitively, involvement of adjacent vestibulocerebellar pathways may have contributed to the patient’s positional symptoms.

An additional feature of this case was the presence of a left internuclear ophthalmoplegia, suggesting involvement of the medial longitudinal fasciculus – despite a negative brainstem MRI. This clinicoradiological discrepancy reflects known limitations of magnetic resonance imaging in the posterior fossa. Acute diffusion-weighted imaging may miss up to 50% of small (< 10 mm) posterior fossa strokes[9]. Furthermore, standard axial averaging can obscure vertically oriented brainstem lesions detectable only on sagittal sequences[10]. A small MRI-occult ischemic lesion affecting the medial longitudinal fasciculus remains a possible explanation.

Previous case reports have described vertebral artery dissection presenting with symptoms mimicking peripheral vestibular disorders^[^11–13^]^. However, many reported patients had associated warning symptoms, such as headache or neck pain^[^11–13^]^. In contrast, our patient presented without pain despite underlying posterior circulation ischemia. Consequently, the complete lack of pain, combined with specific brainstem signs (internuclear ophthalmoplegia) and initially inconspicuous imaging findings, makes this case a rare and diagnostically perilous variation of vascular vertigo.

The optimal antithrombotic strategy for extracranial vertebral artery dissection remains an area of clinical uncertainty, although high-quality evidence from the Cervical Artery Dissection in Stroke Study (CADISS) trial demonstrated no significant difference in stroke recurrence or recanalization rates between anticoagulation and antiplatelet therapies[14]. Current guidelines from the American Heart Association (AHA) and European Stroke Organization (ESO) consider antiplatelets a reasonable and often safer first-line approach^[^15,16^]^. Our patient was treated with aspirin in accordance with current recommendations and showed favorable clinical recovery[14].

## Conclusion

This case highlights that vertebral artery dissection can present as a stroke without typical pain symptoms, devoid of headache or neck pain, and clinically resembling benign vestibular disorders. It illustrates the critical value of the neurological examination – specifically for subtle signs like internuclear ophthalmoplegia – in reclassifying “benign” vertigo as central, even when MRI findings are inconspicuous. Clinicians must maintain a high index of suspicion for vascular etiologies in young patients with acute vertigo, as reliance on pain symptoms or standard neuroimaging alone may lead to missed diagnoses in the posterior fossa.

## Data Availability

The data supporting the findings of this case report are available from the corresponding author upon reasonable request.

## References

[1] Newman-toker DE Hsieh YH Camargo CA. Spectrum of dizziness visits to US emergency departments: cross-sectional analysis from a nationally representative sample. Mayo Clin Proc 2008;83:765–75. 18613993 10.4065/83.7.765 PMC3536475

[2] Saber Tehrani AS Kattah JC Kerber KA. Diagnosing stroke in acute dizziness and vertigo: pitfalls and pearls. Stroke 2018;49:788–95. 29459396 10.1161/STROKEAHA.117.016979 PMC5829023

[3] Lee SH Kim JS. Differential diagnosis of acute vascular vertigo. Curr Opin Neurol 2020;33:142–49. 31789704 10.1097/WCO.0000000000000776

[4] Choi JY Kim JH Kim HJ. Central paroxysmal positional nystagmus: characteristics and possible mechanisms. Neurology 2015;84:2238–46. 25957336 10.1212/WNL.0000000000001640

[5] Kim J Lee H. Vertigo due to posterior circulation stroke. Semin Neurol 2013;33:179–84. 24057820 10.1055/s-0033-1354600

[6] Schievink WI. Spontaneous dissection of the carotid and vertebral arteries. N Engl J Med 2001;344:898–906. 11259724 10.1056/NEJM200103223441206

[7] Gottesman RF Sharma P Robinson KA. Clinical characteristics of symptomatic vertebral artery dissection: a systematic review. Neurologist 2012;18:245–54. 22931728 10.1097/NRL.0b013e31826754e1 PMC3898434

[8] Johkura K. Central paroxysmal positional vertigo: isolated dizziness caused by small cerebellar hemorrhage. Stroke 2007;38. doi:10.1161/STROKEAHA.106.480319 17446419

[9] Saber Tehrani AS Kattah JC Mantokoudis G. Small strokes causing severe vertigo: frequency of false-negative MRIs and nonlacunar mechanisms. Neurology 2014;83:169–73. 24920847 10.1212/WNL.0000000000000573 PMC4117176

[10] Takeshige N Aoki T Sakata K. Sagittal diffusion-weighted imaging in preventing the false-negative diagnosis of acute brainstem infarction: confirmation of the benefit by anatomical characterization of false-negative lesions. Surg Neurol Int 2019;10:180. 31637081 10.25259/SNI_182_2019 PMC6778332

[11] Shah B Manandhar N Paudel R. Funny turns in Young badminton player: a case of cerebellar infarction secondary to vertebral artery dissection. J Adv Intern Med 2021;10:80–82.

[12] Lance S Thomson T. Cerebellar nodulus infarction secondary to vertebral artery dissection. BMJ Case Rep 2019;12:e229876. 10.1136/bcr-2019-229876 PMC6506197 31036743

[13] Cárdenas O Gomez E Marcín M. Bilateral internuclear ophthalmoplegia in a young woman with vertebral artery dissection. European J Case Reports Int Med 2019;6:1. 10.12890/2019_001105 PMC6601697 31293990

[14] Markus HS Levi C King A. for the Cervical Artery Dissection in Stroke Study (CADISS) investigators. Antiplatelet therapy vs anticoagulation therapy in cervical artery dissection: the Cervical Artery Dissection in Stroke Study (CADISS) randomized clinical trial final results. JAMA Neurol 2019;76:657. 30801621 10.1001/jamaneurol.2019.0072 PMC6563567

[15] Kleindorfer DO Towfighi A Chaturvedi S. 2021 guideline for the prevention of stroke in patients with stroke and transient ischemic attack: a guideline from the American heart association/American stroke association. Stroke 2021;52. doi:10.1161/STR.0000000000000375 34024117

[16] Debette S Mazighi M Bijlenga P. ESO guideline for the management of extracranial and intracranial artery dissection. Eur Stroke J 2021;6:XXXIX–LXXXVIII. 10.1177/23969873211046475 PMC8564160 34746432

